# Normalization of wall shear stress as a physiological mechanism for regulating maternal uterine artery expansive remodeling during pregnancy

**DOI:** 10.1096/fba.2021-00019

**Published:** 2021-07-05

**Authors:** Eliyahu V. Khankin, Nga Ling Ko, Maurizio Mandalà, S. Ananth Karumanchi, George Osol

**Affiliations:** ^1^ Department of Medicine Beth Israel Deaconess Medical Center Harvard Medical School Boston MA USA; ^2^ Department of Obstetrics, Gynecology and Reproductive Sciences University of Vermont Larner College of Medicine Burlington VT USA; ^3^ Department of Biology, Ecology and Earth Sciences University of Calabria Rende Italy; ^4^ Department of Medicine Cedars‐Sinai Medical Center Los Angeles CA USA

**Keywords:** endothelium, mechanotransduction, normalization, preeclampsia, pregnancy, rat, vascular remodeling, wall shear stress

## Abstract

Outward remodeling of the maternal uterine circulation during pregnancy is essential for normal uteroplacental perfusion and pregnancy outcome. The physiological mechanism by which this process is regulated is unknown; we hypothesized that it involved the normalization of wall shear stress (WSS). Pregnant Sprague–Dawley rats underwent unilateral ligation of the main uterine artery and vein at the cervical end of the uterus on gestational day 10, thus restricting inflow/outflow of blood into that uterine horn to a single point at the ovarian end; the contralateral sham‐operated side provided an internal control. This procedure alters uterine hemodynamics by increasing WSS, since the entire uterine horn is supplied by one rather than two vessels. Arterial diameter and blood flow velocity values were measured by intravital ultrasonographic pulse‐wave Doppler on gestational day 20 and used to calculate WSS. Although both ovarian artery lumen diameter and blood velocity increased, WSS was similar in both horns. These data support the concept that increased WSS secondary to hemochorial placentation is the primary physiological stimulus for uterine vascular remodeling and that its normalization may be the primary mechanism that regulates the extent of arterial circumferential growth required to maintain placental perfusion. We further hypothesize that shallow spiral artery invasion, such as occurs in preeclampsia, limits the increase in upstream shear stress and results in attenuated remodeling and placental under‐perfusion.

**Abbreviations**
CH: control (sham‐operated) horn
IVS: intervillous space
LH: ligated horn
MUA: main uterine artery
MUV: main uterine vein
NO: nitric oxide
UPBF: uteroplacental blood flow
VSM: vascular smooth muscle
WSS: wall shear stress

## 1 INTRODUCTION

A vital component of the maternal adaptive process during gestation is the growth and remodeling of the uterine circulation. This process, which involves outward expansive hypertrophic circumferential remodeling of arteries and veins, is essential for facilitating the progressive increase in uteroplacental blood flow (UPBF) required to sustain fetal growth and development.^1^, ^2^, ^3^, ^4^ Despite intensive study, the physiological mechanisms that initiate and regulate the process of uterine vascular remodeling are not well understood. Since UPBF increases throughout pregnancy, there must exist a physiological mechanism that balances placental perfusion with the metabolic demands of a growing fetus to avoid underperfusion/ischemia or excessive placental inflow pressures that may damage the fetal villi.^5^ In every mammalian species, including humans, the maternal uterine circulation growth during pregnancy is substantial, with 50%–200% increases in arterial caliber and >10‐fold increases in UPBF by term.^3^, ^4^

Wall shear stress (WSS) has been well‐established as a stimulus for arterial circumferential growth in other regional circulations such as the cerebral and splanchnic.^6^, ^7^ Although the mechanisms are not fully understood, several *in vivo* studies have shown that the remodeling in response to change in WSS does not occur if the endothelial layer is removed.^8^ Several molecules such as transient receptor potential (TRP) and Piezo1 cation channels, as well as some intracellular structures (such as the actin cytoskeleton), are responsive to shear stress and therefore potential mechanotransducers.^9^, ^10^, ^11^

With regard to the uterine circulation during pregnancy, we recently discovered that Piezo1 channels were upregulated during gestation and that they were responsible for intraluminal flow‐induced dilation of isolated rat uterine arteries.^9^ Also, we and others have shown that endothelial nitric oxide is key to the process of gestational maternal uterine vascular remodeling and that this mechanism may be compromised in preeclamptic women.^12^ Hence, one putative mechanism is shear‐induced activation of endothelial Piezo1 channels leading to the entry of cations (particularly calcium), activation of endothelial nitric oxide synthase (eNOS), production and secretion of nitric oxide, and subsequent vasodilation and expansive remodeling. In earlier studies, we ascertained that the influences on remodeling are entirely local and that changes in systemic hormone levels play only a minor role in the process.^3^

What is unknown is how the remodeling process (e.g., its pattern and extent) is regulated and coordinated within the uterine arterial tree. Notably, in preeclampsia, the process of spiral artery invasion is attenuated, resulting in placental underperfusion and the elaboration of antiangiogenic molecules such as sFlt‐1 that act to increase maternal blood pressure, presumably in an effort to drive more blood into the intervillous space (IVS).^13^ If shear stress is a key physiological signal, poor spiral artery remodeling, and placentation would result in lower‐than‐normal shear stress increases in upstream vessels and, hence, attenuated circumferential expansive remodeling. An alternate—or additional—explanation is that it is the processes of mechanosensation and mechanotransduction that are compromised due to the maternal endothelial damage characteristic of PE^14^ and that this attenuates remodeling and limits subsequent placental perfusion.

Here, we hypothesized that the gestational increases in upstream blood velocity and WSS triggered by hemochorial placentation provide an important physiological signal for coordinating the expansive growth of all maternal upstream uterine vessels. The combination of spiral artery invasion and hemochorial placentation, microcirculatory ablation, and placental growth during pregnancy reduces distal resistance and stimulates an increase in WSS in upstream arteries. While the increased WSS may initially trigger arterial dilation and outward remodeling, the consequent increase in arterial lumen diameter would allow flow to be re‐set to a higher volume and velocity but with a similar WSS. Based on this concept, we propose the corollary hypothesis that normalization of shear stress is the physiological mechanism that both determines and limits the extent of arterial remodeling. These hypotheses were tested by altering uteroplacental hemodynamics using a surgical ligation model in pregnant rats. Using a rodent model offers two distinct experimental advantages: (1) the remodeling process is rapid, and the underlying mechanisms are relatively well‐characterized, (2) the bicornuate rodent uterus offers the benefit of each animal providing its own internal control (the contralateral horn) since the vascular supply to each horn is independent of the other.

## 2 MATERIALS AND METHODS

### 2.1 Animals

Pregnant Sprague–Dawley rats (*n* = 21; gestational day 8) were obtained from Charles River laboratories and singly housed at the Animal Research Facility at the Beth Israel Deaconess Medical Center, which is fully accredited for the Association for the Assessment and Accreditation of Laboratory Animal Care International. Feed and water were provided ad libitum. All experiments and procedures were approved at the Beth Israel Medical Center.

### 2.2 Surgical protocols

**FIGURE 1:**
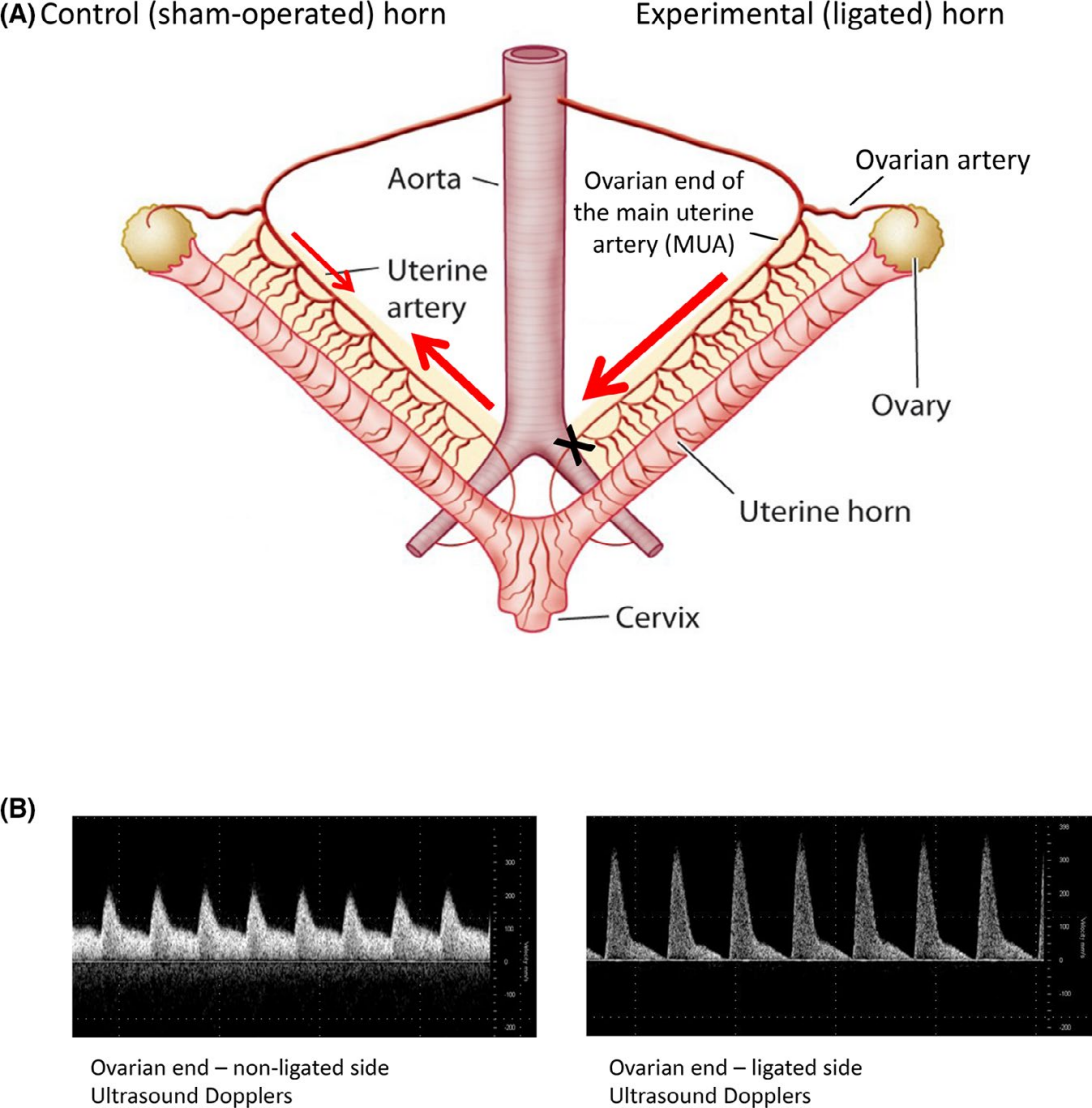
(A) Schematic of the surgical approach. (B) Representative ultrasound images taken from the main uterine arteries from the control and ligated uterine horns, respectively

In rodents and humans, the uterus is normally supplied by blood from the two main uterine arteries (MUAs) which originate from the anterior division of the internal iliac arteries, and from the ovarian arteries, which branch off of the abdominal aorta below the renal arteries. Because the ovarian and uterine arteries anastomose to form a loop, the inflow of blood to the uterus is normally bidirectional.^2^ By ligating the main uterine artery and vein at the cervical end in one horn (Figure 1A), we were able to create a single point of inflow from the ovarian artery.

Creating a predicable flow pattern and enlarging the perfusion territory increases shear stress within the ovarian end of the MUA and results in its significant enlargement.^15^ Conversely, in the opposite control (sham‐operated) horn, inflow is bidirectional, with a greater proportion coming from the cervical end rather than the ovarian end.^2^

Surgeries were performed under general anesthesia (inhaled isoflurane) by abdominal laparotomy on day 10 of gestation, before the onset of significant remodeling (in the rat, implantation does not occur until gestational day 5 or 6, as described elsewhere).^15^ Ten days later (on gestational day 20/22), rats were again anesthetized, the abdominal cavity opened, pregnant uteri, and its vasculature exteriorized for measurement of uterine artery flow using small animal ultrasonographic pulse wave Dopplers from Vevo 2100 (VisualSonics). The animal was kept on the warmed table of the Vevo 2100 system and had a rectal temperature probe inserted, allowing maintenance of body temperature within normal physiological limits (36.0°C–37.5°C). External infrared lamp warmers were used as needed to supplement temperature maintenance measures in order to maintain body temperature within the aforementioned physiological limits. The exposed parts of the uterus and mesometrial arcade were covered either with warm gel for ultrasonography (temperature of the gel maintained at the same physiologic limits described above)—this was done at the point where ultrasonography probe was placed for measurements—or wrapped in high grade surgical woven sterile gauze pads soaked in the warm physiological buffer (buffer temperature also maintained at 36°C–37.5°C) at the exposed parts of the uterus where no gel was applied. A 48 MHz transducer operating at 100 fps was used to image the uterine artery. In Doppler mode, pulsed repetition frequency was set between 4 and 48 kHz to detect low to high blood flow velocities, respectively. A 2–5 mm pulsed Doppler gate was used, and the angle of the Doppler beam and the vessel was recorded and kept <60°. Waveforms were saved for later offline analysis. All scans were performed by the same operator (EK). Anatomical position and color Doppler mode were utilized to ensure correct identification of vascular structures being studied. The ultrasound parameters obtained included animal heart rate, uterine artery pulse wave Doppler, and uncompressed arterial diameters. After identifying the ovarian end of the MUA, based on the observed uterine anatomy, and utilizing color Doppler mode for verification, the cross‐sectional diameter of the MUA (before the first arcuate artery branch) was measured and recorded using M‐Mode. Following that, peak systolic velocity (PSV) and end‐diastolic velocity (EDV) were measured over five cardiac cycles that were not affected by the motion artifacts caused by maternal breathing, and the results were averaged and reported as mean velocity expressed in cm/sec. (Figure 1B).

Following euthanasia, the uterus and its vasculature were removed *en bloc* and shipped to Vermont in tubes filled with physiological buffer pack on ice. Upon receipt, each uterus was positioned with pins in a silicone‐coated Petri dish containing isotonic buffer to expose the course of the uterine arcade. The unstressed lumen diameter of the main uterine artery (MUA) was measured at the approximate midpoint of the mesometrial arcade with a stereomicroscope (Zeiss). The correlation between directly measured MUA diameter and measurements made with ultrasound imaging was *r* ^2^ = 0.72.

### 2.3 Measurements and statistical analysis

Shear stress is a product of viscosity and velocity divided by vessel radius^3^. Wall shear stress (*Ƭ* in formula, abbreviated WSS in text) was calculated as: *Ƭ* = 4*ƞQ*/*πr* ^3^, where *ƞ* is the viscosity (assumed to be constant), *Q* is the velocity, and *r* is the radius. Values for *Q* and *r* were obtained from the in vivo ultrasound measurements of the exteriorized main uterine artery and vein at the ovarian end of each horn. Calculations were made for both ovarian arteries, the ovarian end of a uterine horn. Results were expressed in absolute and relative (percent change) terms. The prediction was that a larger perfusion territory would augment blood flow and outward circumferential remodeling of upstream vessels, but that WSS at the ovarian end of the MUA would be comparable in the ligated versus control horn.

All measurements are reported as mean ± SEM. Statistical analysis utilized the paired Student's *t*‐test comparing data from the ligated versus control horn within each animal and *p* ≤ 0.05 was considered significant.

## 3 RESULTS

As reported in an earlier study,^15^ MUA ligation did not alter the reproductive outcome in terms of the pup and placental weights, or incidence of resorptions (data not shown), indicating that the vasculature was indeed able to adapt to the stress of having to perfuse an increased perfusion territory from a single point of inflow.

**FIGURE 2:**
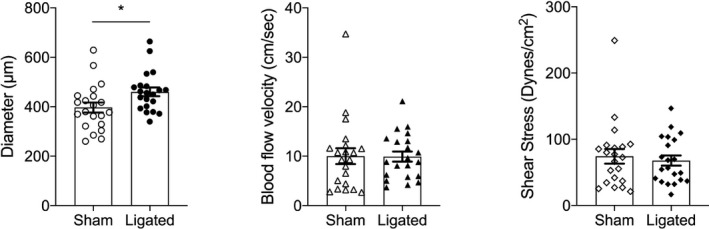
Summary of main uterine artery data from the control (sham) and ligated uterine horn showing changes in both diameter (left panel) and blood flow velocity (middle panel). Calculated wall shear stress is depicted in right panel. Data are reported as mean ± SEM; *n* = 21 animals. * = *p* < 0.05

A summary of the ovarian end MUA diameter, blood flow velocity, and WSS for all animals (*n* = 21) is shown in Figure 2. Although there was a significant increase in ovarian end MUA diameter in the ligated versus control horn, there was no statistical change in blood flow velocity or WSS. There was however, considerable variability in the extent of vascular remodeling relative to the control horn. We interpreted the absence of remodeling as being indicative of incomplete ligation due to a rerouting of the blood through arcuate arteries, which sometimes occurs and is dependent on the vascular architecture of each individual animal. This could not be predicted at the time of surgery since vessels are small and often obscured by periarterial fat, but became visually evident by gestational day 20. An absence of remodeling may also be due to incomplete ligation.

**FIGURE 3:**
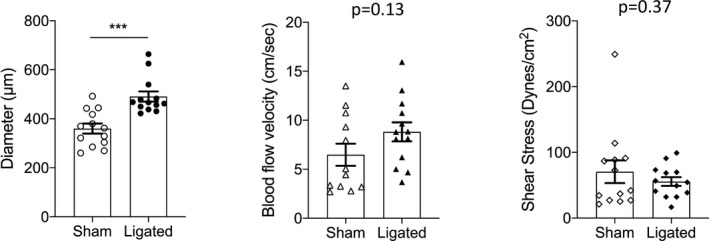
Summary of main uterine artery data from a remodeled subset of animals (*n* = 13) in control (sham) and ligated uterine horn showing changes in both diameter (left panel), blood flow velocity (middle panel), wall shear stress (right panel) is depicted. Data are reported as mean ± SEM; *** = *p* < 0.001

To address this limitation, we performed an additional analysis in a subset of animals (*n* = 13) that showed luminal enlargement relative to the control horn (Figure 3), because we did not want to bias the data by measuring diameters and flow velocity in vessels that did not appear to remodel (and which would thus favor the WSS normalization hypothesis).

**FIGURE 4:**
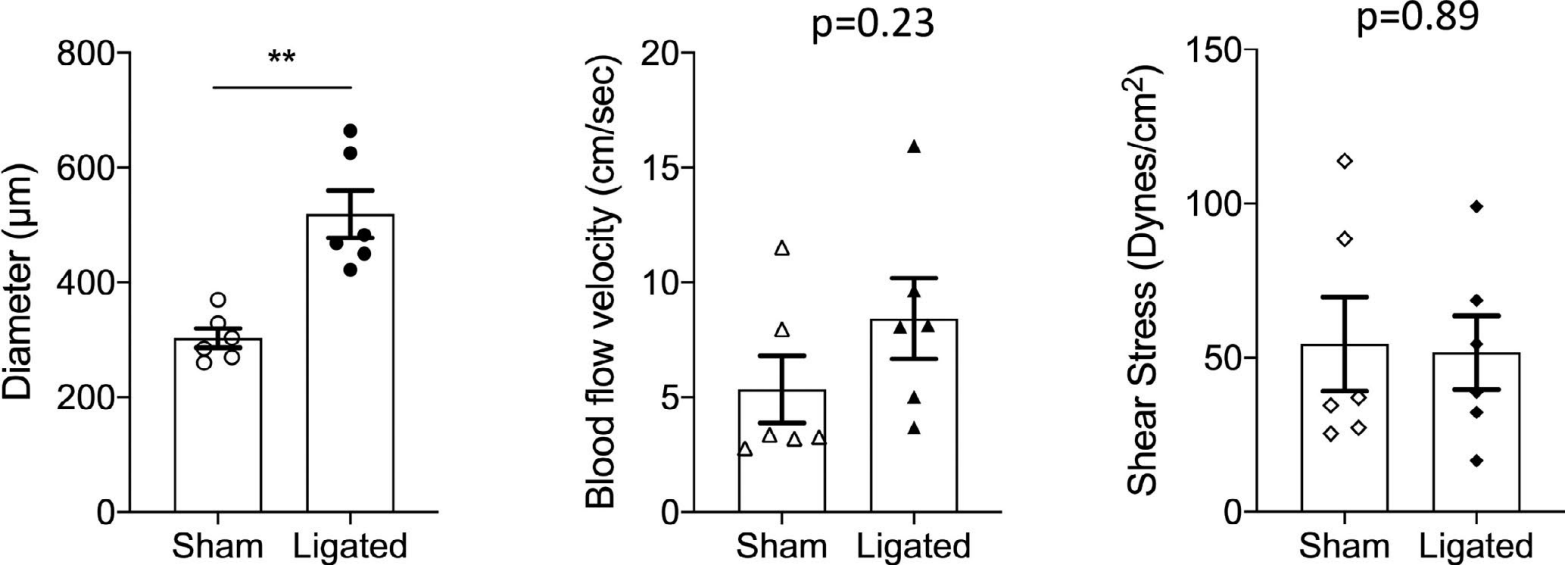
Summary of main uterine artery data from the maximally remodeled subset of animals (*n* = 6) in control (sham) and ligated uterine horn showing changes in both diameter (left panel), blood flow velocity (middle panel), wall shear stress (right panel) is depicted. Data are reported as mean ± SEM; ** = *p* < 0.01

We then sampled the six vessels that showed the greatest extent of remodeling, reasoning that this dataset would be most revealing of the adaptive response. As shown in Figure 4, lumen diameters on the ligated side were increased by 71 ± 9% (*p* = 0.001), and velocity was also increased approximately 105 ± 64%, although this difference did not attain significance (*p* = 0.23), most likely due to the small sample size. WSS, on the other hand, was virtually identical in the ligated versus sham‐operated horn (*p* = 0.89).

## 4 DISCUSSION

**FIGURE 5:**
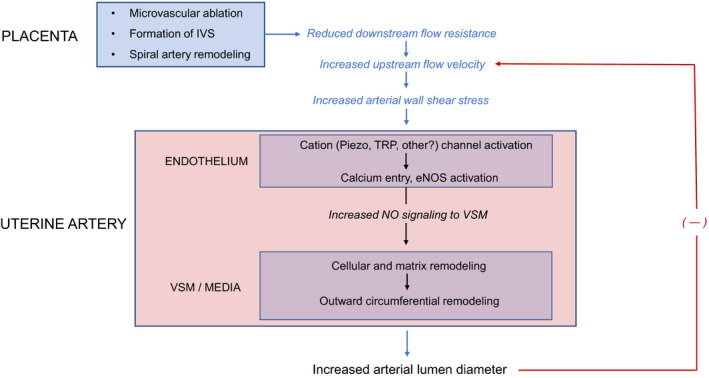
Conceptual drawing illustrating the wall shear stress (WSS) normalization hypothesis. Briefly, a reduction in downstream flow resistance due to placentation leads to an acceleration of blood in upstream arteries, increasing upstream flow velocity, and arterial WSS. Subsequent outward circumferential remodeling of these vessels allows the accommodation of increased flow at a lower velocity and larger lumen diameter. As a result of these changes, WSS is reduced to its original value. This negative feedback loop (―) ultimately regulates the extent of remodeling and maintains placental perfusion at an adequate level

By using an ultrasound‐based technique for measuring uterine artery lumen diameter and blood velocity values, we were able to calculate WSS, and found that, unlike diameter and blood flow velocity, it was quite similar in the ligated versus control (sham‐operated) horn. Based on this finding, we propose that WSS normalization offers a means for coordinating the expansion of the uterine vascular tree in a way that prevents placental under‐ or over‐perfusion. As the placenta develops and grows, distal resistance progressively decreases, resulting in an acceleration of blood in upstream vessels. This leads to an increase in WSS, and a ‘corrective’ response on the part of the vasculature through vasodilation and growth ‐ processes that, in turn, reduce WSS to its normal set point (Figure 5).

As we, and others have shown, endothelial nitric oxide release is the active principle that increases arterial diameter through a combination of vasodilation and expansive circumferential remodeling. Our most recent data suggest that Piezo1 cation channels may be the primary molecular mechanotransducers of WSS into nitric oxide production via calcium entry and eNOS activation.^9^

There are several limitations to these findings that deserve consideration. First, blood is a non‐Newtonian fluid. Therefore, using the conventional WSS formula provides values that may not reflect true shear stress at the endothelial surface. The velocity profile within a blood vessel is often depicted as being parabolic, with the highest flow velocity in the center. Hence, we can only approximate true WSS based on the velocity measurements obtained from the small animal micro‐ultrasound. Moreover, because of the pulsatility of pressure within a vessel, WSS is oscillatory. Thus, while they may be state‐of‐the‐art, ultrasound measurements do not allow us to measure true velocity at the endothelial surface, or to resolve changes in velocity within each cardiac cycle. Second, because we were unable to obtain accurate transabdominal ovarian artery measurements in the rat, animals were anesthetized and laparotomized to expose the uterus. Although each rat was placed on a heating pad, some evaporative cooling inevitably occurred, and we do not know whether the application of ultrasound gel onto the uterine mesometrium had any effect on arterial diameter or tone. Third, the exclusion of data from animals in whom ovarian arteries did not remodel (or remodeled less than previously documented) reduced the *n* value and introduced some experimental bias. At the same time, if the hypothesis is not correct, focusing on the subgroup of animals that showed the greatest extent of arterial remodeling confers a negative bias with respect to hypothesis evaluation, i.e., likely to bias the results against rather than for the hypothesis, since the differences in WSS would likely be greatest, not least. As shown in an earlier study using this surgical ligation model, reproductive outcomes such as pup weights, pup numbers, and placental weights are not altered^15^ and therefore our model is less useful to study the role of MUA remodeling in placental disorders. Finally, we also did not evaluate systemic blood pressures in this model and are therefore unable to evaluate the relevance of these findings to the pathogenesis of preeclampsia.

However, two clinical scenarios related to placentation abnormalities deserve consideration. The first is that poor spiral artery invasion (which is often noted in preeclampsia or idiopathic fetal growth restriction) may limit the increase in upstream WSS by not providing a sufficient decrease in distal resistance, and result in attenuated maternal arterial remodeling and placental under‐perfusion. In this scenario, the WSS normalization mechanism works normally, but the extent of remodeling is limited by poor placentation. An alternate explanation is that, even when spiral artery invasion is reasonably adequate, maternal endothelial damage incurred during disease progression compromises the WSS sensing and/or transducing mechanisms. For example, insufficient upregulation of Piezo1 channels on the luminal surface of the endothelium, or a post‐channel attenuation of mechanotransduction pathways involving second messenger and signal transduction pathways (such as eNOS/NO signaling) could, in turn, impair maternal uterine vascular remodeling mechanisms and limit the delivery of blood to the placenta. These two possibilities are not mutually exclusive and may well both occur. It would be most interesting and insightful to measure arterial diameter, flow velocity, and WSS in pregnant women with or without preeclampsia. In one earlier transvaginal ultrasound study, thus far published only in abstract form,^16^ uteroplacental blood flow in healthy women increased >1100% by week 32 of pregnancy (relative to the luteal phase) while WSS only increased by 20%, a value that is well within the margin of error for ultrasound measurements. These data in humans are striking and support the WSS normalization hypothesis.

In summary, we provide in vivo evidence that WSS is an important physiological set point for regulating and coordinating uterine arterial growth and remodeling. Additional studies are needed to explain why this process may be compromised in gestational diseases such as preeclampsia, where attenuated placentation and/or maternal endothelial damage may diminish the increase in WSS or the cellular response to a normal increase in WSS, resulting in a smaller‐than‐normal arterial caliber, placental under‐perfusion, and a progression in disease severity.

## AUTHOR CONTRIBUTIONS

All the experimental data included in this paper were generated by Drs. Khankin, Ko, and Mandala. Drs. Karumanchi and Osol provided contributions to the conception and design of the study. All five authors contributed to the interpretation and writing of the paper.
